# Age differences in sustained attention tasks: A meta-analysis

**DOI:** 10.3758/s13423-021-01908-x

**Published:** 2021-03-26

**Authors:** Antonino Vallesi, Virginia Tronelli, Francesco Lomi, Rachele Pezzetta

**Affiliations:** 1 Department of Neuroscience & Padova Neuroscience Center, University of Padova, 35131 Padova, Italy; 2 IRCCS San Camillo Hospital, Venice, Italy; 3 Department of General Psychology, University of Padova, Padova, Italy

**Keywords:** Sustained attention, Vigilance, SART, Cognitive aging, Go/no-go, Motor inhibition

## Abstract

Many aspects of attention decline with aging. There is a current debate on how aging also affects sustained attention. In this study, we contribute to this debate by meta-analytically comparing performance on the go/no-go Sustained Attention to Response Task (SART) in younger and older adults. We included only studies in which the SART had a low proportion of no-go trials (5%–30%), there was a random or quasirandom stimulus presentation, and data on both healthy younger and older adults were available. A total of 12 studies were suitable with 832 younger adults and 690 older adults. Results showed that older adults were slower than younger adults on go trials (*g* = 1, 95% CI [.72, 1.27]) and more accurate than younger adults on no-go trials (*g* = .59, 95% CI [.32, .85]). Moreover, older adults were slower after a no-go error than younger adults (*g* = .79, 95% CI [.60, .99]). These results are compatible with an age-related processing speed deficit, mostly suggested by longer go RTs, but also with an increased preference for a prudent strategy, as demonstrated by fewer no-go errors and greater posterror slowing in older adults. An inhibitory deficit account could not explain these findings, as older adults actually outperformed younger adults by producing fewer false alarms to no-go stimuli. These findings point to a more prudent strategy when using attentional resources in aging that allows reducing the false-alarm rate in tasks producing a tendency for automatic responding.

The ability to maintain the focus of attention on a task over time is known as sustained attention or vigilance, and it is a fundamental component of normal cognitive capacities. Indeed, without this ability, many other cognitive functions would be compromised (Parasuraman, 1998). Given its importance for general cognitive functioning, sustained attention has been investigated in many studies.

One of the first experimental tasks used to study sustained attention dates back to the 1950s and was used to evaluate vigilance in the British Air Force (Mackworth, 1948). The original device—known as the “Mackworth Clock”—was similar to a watch with a pointer moving with short jumps. Double jumps occurred at irregular intervals, and the task was to respond to them by pushing a button. The overall task duration was about 2 hours. At first, this might be an easy task, and one would rarely make mistakes. With time on task, however, it can become harder and harder to maintain the attentional focus and accuracy starts to decrease.

This task was the starting point for many studies on sustained attention. Over the years, new tasks were developed in which the participant has to monitor a continuous flow of stimuli for a prolonged period and has to respond to rare target stimuli. These types of tasks have recently been defined as “traditionally formatted tasks” (TFTs; Stevenson et al., 2011). In this case, the vigilance decrement is the index of deterioration of sustained attention, characterized by a decrease in accuracy and/or an increase in reaction times (RTs) with time on task. The duration of TFTs varies between studies (from 150 s to 2 h), but the average duration is about 30–45 minutes (Staub et al., 2013).

Another type of task aimed at investigating sustained attention is the Sustained Attention to Response Task (SART; Robertson et al., 1997). The original SART introduced by Robertson et al. (1997) is a no-go task with a quasirandom presentation of digits from 1 to 9, in which the participant has to respond to all the digits except for 3, which is the no-go target. Digits are presented for 250 ms, followed by a 900-ms mask. The task takes about 4 minutes. The no-go trials represent only 11% of total trials, in order to favour an automated response to go trials. Hence, contrary to a TFT, the SART requires one to withhold the response to targets and to respond to nontargets. Robertson and colleagues argued that sustained attention to the task would be taxed more heavily if the automatic response was directed to nontarget stimuli. Indeed, the active-controlled processing could be activated more to overcome the prepotent automatic response at the onset of the rare target. In this sense, the commission errors (i.e., response to target) are the main indicator of the impaired sustained attention ability. The SART is more sensitive to sustained attention deficits than are traditional vigilance tasks (Staub et al., 2013) and seems to have a higher ecological validity: Commission errors are indeed positively correlated with a tendency to report everyday cognitive errors (Manly et al., 1999; Robertson et al., 1997), and more specifically, attention-related everyday cognitive errors (Cheyne et al., 2006).

Sustained attention is essential for functioning in everyday life; thus, it is important to understand how it changes across the adult lifespan, and in particular with aging. Several studies reported that older adults showed longer RTs and fewer errors on sustained attention tasks than younger adults (e.g., Brache et al., 2010; Carriere et al., 2010; Grandjean & Collette, 2011; Heilbronner & Münte, 2013; Hsieh et al., 2016; Jackson et al., 2013; Jackson & Balota, 2012; Kousaie et al., 2014; McVay et al., 2013; Mioni et al., 2019; Staub et al., 2014c; Staub et al., 2015). Longer RTs could be in line with an age-related processing speed deficit (Salthouse, 1996), which has been attributed, among other factors, to the reduction in white matter integrity associated with aging (Salthouse, 2017). However, the longer RTs and the difference in the amount of errors also suggest a conservative strategy to compensate for their poor response inhibition (Staub et al., 2013): in other words, older adults could be more cautious in responding on go trials to avoid errors on no-go trials. Although many studies show higher performance in terms of accuracy for older adults on go/no-go tasks, there are contrasting results reporting no age-related differences or even better performance in younger adults (e.g., Cassarino et al., 2019; Harty et al., 2013; Hong et al., 2014; Hsieh et al., 2016a, 2016b; Langenecker et al., 2007; Lin et al., 2018; Lucci et al., 2013; McAvinue et al., 2012; Nielson et al., 2002; Rush et al., 2006; Vallesi & Stuss 2010; Vallesi, 2011; Vallesi et al., 2011; Zavagnin et al., 2014).

To deal with these issues, the objective of the present meta-analytical study is to contribute to the debate on SART performance in cognitive aging. To this end, we selected the studies that used a cross-sectional design involving participants from 18 to 95 years of age.

The first aim was to determine the difference between older and younger adults on SART performance, above all in terms of accuracy on no-go trials. This variable indicates the ability to avoid a commission error (i.e., the capacity to inhibit the response). Indeed, calculating the accuracy on no-go trials was useful in investigating whether the inhibition capacities in older adults are preserved (Rey-Mermet & Gade, 2018) or impaired (Hasher & Zacks, 1988). Further, previous studies found that the stimulus evaluation in younger adults decreases with time on task, as compared with older adults, in whom the evaluation processes become even more controlled as the task advances. This suggests that younger adults might adopt a more automatic behavior, rather than a careful and controlled strategy (Carriere et al., 2010; Staub et al., 2015). Thus, in line with previous reports, we expected that response automatization could occur in younger adults, and consequently it could increase the likelihood of committing errors on no-go trials (Staub et al., 2015). Conversely, older adults could adopt a high degree of control over the motor system, enabling them to reach a good level of performance (Staub et al., 2015).

Indeed, some studies (Jackson et al., 2013; Jackson & Balota, 2012; Staub et al., 2014b, 2014c; Staub et al., 2015) reported a reduction in self-reported mind-wandering in older adults compared with younger ones while performing the SART. This may be attributable to older adults finding the SART more difficult and/or more engaging than do younger ones (Jackson et al., 2013; Jackson & Balota, 2012; Staub et al., 2014b, 2014c; Staub et al., 2015). These age differences may have resulted in more effort, and therefore less mind-wandering and a higher degree of control over the motor system in the older group (Jackson & Balota, 2012). A high degree of motor control could also be associated with the increase of RTs in older adults: they may prefer to be slower in order to be more careful and cautious in responding (speed–accuracy trade-off; Staub et al., 2013). For this reason, beside the screening of RTs in go trials in younger and older adults, we considered necessary to also analyze the posterror slowing (PES)—namely, the prolonged RT that is observed after the commission of an error. Indeed, several studies found that RTs after a commission error on no-go trials were increased more in older adults than in younger ones (Jackson & Balota, 2012; McVay et al., 2013; Staub et al., 2014c)*.*

One of the main accounts for PES suggests that this effect reflects the implementation of cognitive control to improve subsequent performance (Danielmeier & Ullsperger, 2011). Cognitive control refers to processes that allow information processing of current goals and support flexible, adaptive, and complex responses. Hence, the increased PES in older adults may be indicative of a decline in cognitive control ability—that is, a difficulty in reestablishing the task set after an error has been made (Jackson & Balota, 2012). Moreover, the age difference in PES could be due to the engagement of a type of reactive thought process, also called “task-related interference” (Smallwood et al., 2004): Older adults could be more conscientious, and hence increase their self-assessment of performance after an error, thereby producing prolonged RTs (Jackson & Balota, 2012; Staub et al., 2013). The two hypotheses are not mutually exclusive.

Finally, we also analyzed the accuracy on go trials to evaluate the ability not to make an omission error. We expected to find no age-related differences (Carriere et al., 2010; Hsieh et al., 2016; Jackson et al., 2013; Jackson & Balota, 2012; McAvinue et al., 2012; McVay et al., 2013; Mioni et al., 2019). Indeed, this type of response should be simpler than no-go trials, as we chose to include only studies with a higher percentage of go trials. The second aim of this meta-analytical study was to investigate how performance varies over time in older and younger adults. Based on some of the reported findings, we hypothesized a better preservation of performance over time in older adults than in younger ones (Brache et al., 2010; Staub et al., 2014a, 2014b, 2014c; Staub et al., 2015). The more controlled response strategy in older adults could lead them to maintain a stable level of performance in the go/no-go SART over the course of the task. We also checked whether older adults’ performance is associated with increased fatigue over time.

## Method

The meta-analysis is reported according to the Preferred Reporting Items for Systematic reviews and Meta-Analyses (PRISMA; Liberati et al., 2009). Each of the recommended steps (search and eligibility criteria, study selection, data extraction and analysis) were made independently by two authors; results were compared, and possible disagreements were resolved by discussion and consensus with a third author.

### Eligibility criteria

The following inclusion criteria were used to select articles for the meta-analysis:

- Using the Sustained Attention to Response Task (SART; Robertson et al., 1997) or a modified SART version. In the latter case, we included only those works that used paradigms that adhere to the main parameters of the Robertson’s task, such as the presence of a single no-go trial type, random or quasirandom presentation of stimuli, a higher proportion of go trials (i.e., 70%–95%) than no-go trials (i.e., 5%–30%) and instructions emphasizing equally speed and accuracy. Only studies with a lower percentage of no-go than go were chosen to reflect the criteria identified in Mackworth's (1956) review about the nature of classic vigilance tests. According to this author, there are two types of vigilance: one is needed throughout a long test to detect the occasional significant stimuli among many others presented at a slow pace, and the other one is necessary during a short test to detect rare signals among many other rapidly presented stimuli (Mackworth, 1956). We chose the second type because it is closer to more recent definitions of sustained attention (Leclercq, 2002). Furthermore, tasks that adopt no-go stimuli as targets, considered as more difficult than TFTs (Robertson et al., 1997), could be more sensitive to age-related differences.
- Inclusion of healthy samples for younger (about 18–35 years old) and older adults (60 years old and over).
- Enough statistical information, such as means or medians, standard deviations (*SD*) or ranges, separately for the younger and older adults of the whole sample, or *t* or *F,* in order to calculate the differential effect size and perform the meta-analysis.

### Information sources

A systematic literature search was carried out using PubMed, PsycINFO, and Scopus in order to retrieve relevant articles. Further, we checked the references in the selected articles and additional studies on the SART from different sources to find other potentially relevant articles.

### Search strategy

The search for eligible studies was carried out between March and April 2020. Then, an update was performed December 20–21, 2020, but no additional suitable studies were found. The literature search was performed using the conjunction of the following terms: (“older adults” OR “elderly” OR “aging” OR “ageing” OR “cognitive aging” OR “cognitive ageing” OR “normal aging” OR “normal ageing”) AND (“SART” OR “Sustained Attention to Response Task”). All terms were searched both as a keyword within the text and as a word belonging to the title and/or abstract. No restriction on publication date range was applied and only published works with an English version available were considered.

### Study selection

The relevant material was searched through databases, with the strategy explained above, or through other sources (e.g., citations of the articles obtained by database search). The relevance and eligibility of articles were evaluated using a hierarchical approach. The total sum of papers was first assessed for duplicates. Then, the papers were screened on the basis of title and abstract, and those that did not meet the inclusion criteria were excluded. The remaining articles were finally examined in more depth—that is, by reading the full manuscript—and those that met the inclusion criteria were included in the meta-analysis.

When a potentially eligible paper did not provide some necessary information to perform the analyses, the corresponding author was contacted via email. For example, when the study did not stratify the whole sample based on age, we directly contacted via email the authors of the article to ask for the data separately for older and younger adults. If we did not get an answer or the requested information could not be found, that study was discarded.

Before analyzing each variable taken into consideration, some clarifications must be made on some of these included studies:

- The study by Carriere et al. (2010) reported the age groups by decade; hence, only the third decade (for the group of younger adults) and the seventh-plus decade (for the group of older adults) were included in the present meta-analysis, since the age of the other groups was out of our interest range.
- Three studies included different experiments (Jackson et al., 2013; Jackson & Balota, 2012) and/or different conditions within the same experiment (Jackson et al., 2013; Kousaie et al., 2014), involving different participants; therefore, these experiments and conditions were divided and analyzed as independent.
- McAvinue et al. (2012) reported two SART conditions: a random condition, in which the digits appeared in a random order, and a fixed one, in which there was a fixed sequence from 1 to 9. Only the random condition was taken into account as it resembles Robertson’s version. In addition, only the age groups 20s and 30s (for the group of younger adults) and the age groups 60s and 70s (for the group of older adults) were taken into consideration, since the age of the other groups was out of our interest range.
- The study by McVay et al. (2013) assigned participants to two conditions based on the SART version, and we only considered Robertson’s one. The other version was excluded because the participants had to respond to targets, which were 11% of total trials. Hence, like in a TFT, the inhibition of the response did not refer to rare stimuli, but to frequent ones (89%). We contacted the authors in order to obtain the sample size and the performance variables of the standard SART condition, separately for older and younger adults. The authors kindly provided us with the sample size and accuracy on go and no-go trials.
- The study by Hsieh et al. (2016) investigated cognitive performance on the SART after a reading session and an acute resistance exercise session. Since the former was considered as the baseline in that study, we decided to include only the “reading” condition in the meta-analysis.
- In the study by Cassarino et al. (2019), the SART was administered before and after viewing images of natural or urban environments. Therefore, only the SART variables concerning the baseline condition were included.
- We contacted Dr. Mioni for more information on her study data (Mioni et al., 2012). She kindly provided us with another article (Mioni et al., 2019), since the article found by us was a conference proceeding. Moreover, she provided us with the RTs for each trial of each participant and the mean and standard deviation of commission errors and omission errors separately for younger and older adults.

### Data collection process

The meta-analysis was performed using Meta-Essentials software (Suurmond et al., 2017), in particular, the “Differences Between Independent Groups—Continuous Data” workbook, since the main outcome of interest was the mean difference between younger and older adults. All statistical information necessary for performing the meta-analysis was extracted from the retrieved articles, including sample size, means and standard deviations, separately for younger and older adults, or *t* or *F,* so that effect sizes could be calculated or at least estimated. When not directly reported in the text, statistical information was retrieved from plots using WebPlotDigitizer, a software freely available on the internet, which allows to extract numerical data from images (Rohatgi, 2019).

### Data items

Only dependent variables reported by at least five studies were subjected to meta-analysis:

#### RTs (in ms) on correct go trials

**Fig. 1:**
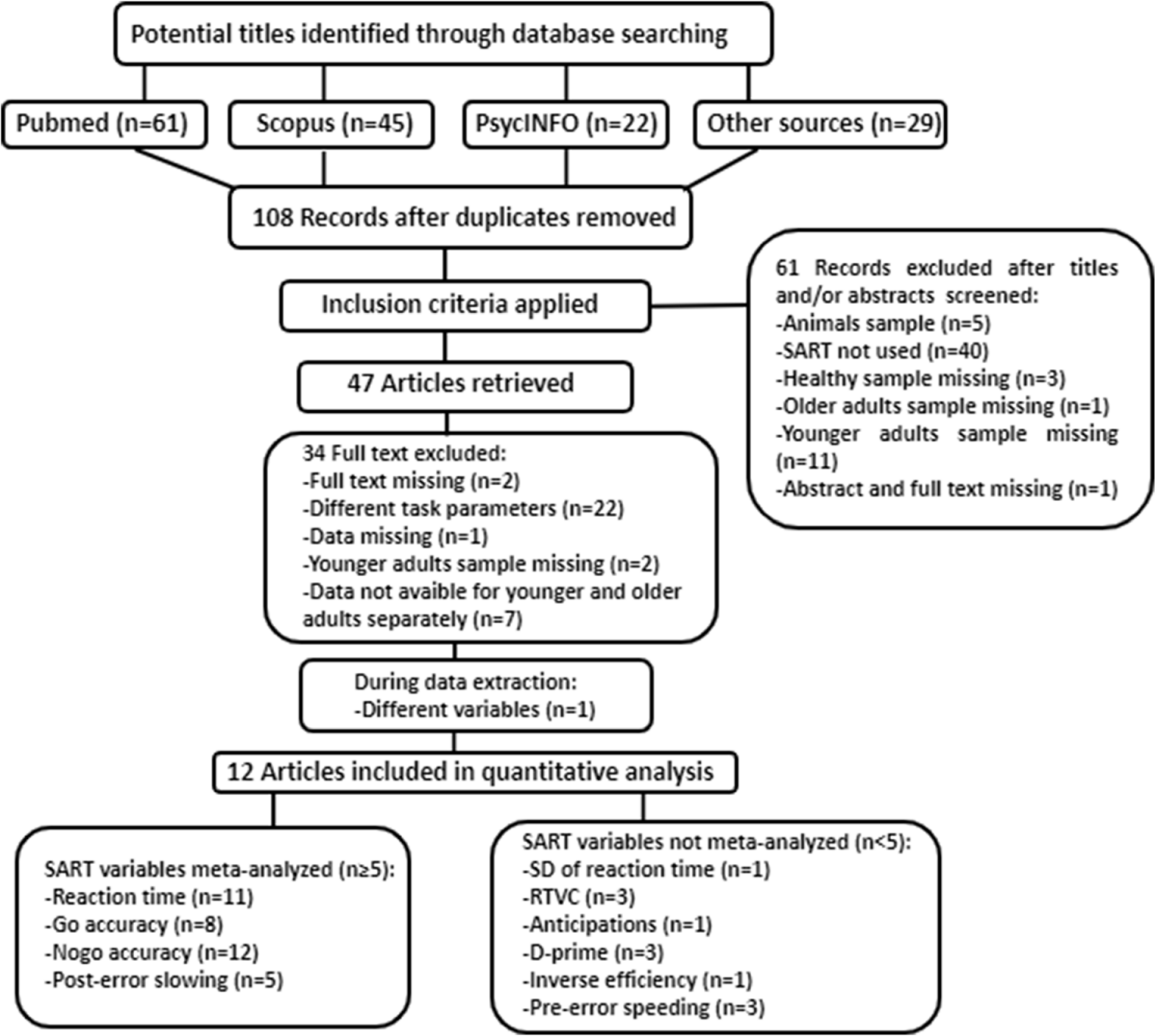
PRISMA flow diagram of the retrieved articles, evaluated according to the inclusion/exclusion criteria and included in the analysis

**Fig. 2:**
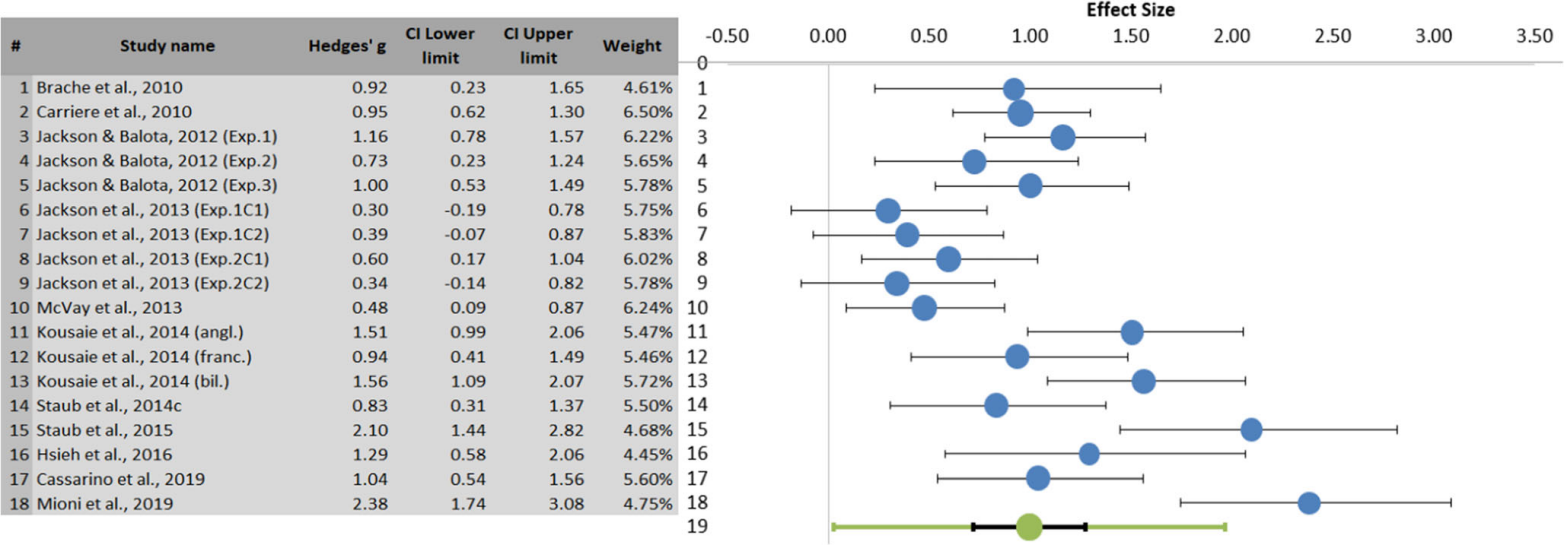
Left: Summary results of the meta-analysis regarding RT differences between younger and older adults, including Hedges’ *g,* confidence interval (CI), and relative weight of each study. The weight was computed as the inverse of the within-study variance with an additive estimate of the between-studies variance (*T*^2^) based on the DerSimonian-Laird method (Van Rhee et al., 2015), since a random effects model was used. Right: Forest plot showing the effect size (in blue) of each study with its confidence interval (in black) and the combined effect size (in green) with its confidence interval (in black) and its prediction interval (in green). The larger the blue dot, the higher the study weight. The positive effect size shows longer RTs in older adults than in younger adults. (Color figure online)

The amount of time taken to respond to routine go stimuli. Eleven articles (Brache et al., 2010; Carriere et al., 2010; Cassarino et al., 2019; Hsieh et al., 2016; Jackson et al., 2013; Jackson & Balota, 2012; Kousaie et al., 2014; McVay et al., 2013; Mioni et al., 2019; Staub et al., 2014c; Staub et al., 2015), for a total of 18 substudies taken separately, were considered in the analysis of correct RTs to go trials. The study by McVay et al. (2013) did not report the RT standard deviation, and therefore the *t* value was considered. The studies by Staub et al. (2014c), Staub et al. (2015) and Cassarino et al. (2019) did not report in the text the mean and standard deviations values of the RTs, so we obtained these data from the graphs shown in these articles (their Fig. 2, Fig. 1, Fig. 2, respectively) with the WebPlotDigitizer program. In the studies by Staub and colleagues the mean and standard deviation were reported separately for the three periods in which the task was subdivided, so we made an average of the three blocks. However, in the Staub et al.’s (2014c) graph, confidence intervals (95%) were reported instead of standard deviations, so the standard deviation was obtained through the formula $SD=\frac{ME}{t_{.025,n-1}}\times \sqrt{n}$ (*ME* = Error Margin; *n* = sample size; *t*_0.025, *n* − 1_= critical value corresponding to an area of .025 in each tail for *n*-1 degrees of freedom). Also, in the Cassarino’s graph there were standard errors instead of standard deviations of RTs, so the latter were obtained through the formula (*SE* = standard error).

#### Posterror slowing (PES; in ms)

It is often quantified as the difference between the mean RTs on the trials immediately following a commission error on no-go trials and the mean RTs on the trials immediately following a correct no-go trial (Danielmeier & Ullsperger, 2011). Three articles (Jackson & Balota, 2012; McVay et al., 2013; Mioni et al., 2019), which included five substudies taken separately, were considered in the analysis of PES. In this case, we only considered the interaction results of the 2 × 2 analysis of variance (ANOVA), with no-go trial response (correct vs. incorrect) as the within-subjects factor and age group (younger vs. older) as the between-subjects factor on go RTs right after no-go trials. Importantly, raw RTs had to be transformed (i.e., into *z*-scores) to account for the age-related generalized slowing. Hence, one study (Staub et al., 2014c) was excluded because, although the authors reported data on PES, they did not apply any kind of transformation on RTs. Among the selected articles, two reported standardized RTs (zRTs) for this analysis (Jackson & Balota, 2012; McVay et al., 2013); for the other study (Mioni et al., 2019), the main author kindly provided us with the necessary data to perform this transformation. Therefore, RT for each go trial was first *z*-transformed for each subject by using this formula: z$RT=\frac{RT- mean\  RT}{SD}$, where *RT* is the raw reaction time at a specific go trial, and *mean RT* and *SD* are the within-subjects mean and standard deviation of go RTs. Then, mean zRT after no-go trials was used as a dependent variable for the 2 × 2 ANOVA mentioned above, and the interaction result was considered for the analysis. Two older adults had to be excluded from this analysis, since they did not have any post-no-go error RTs available.

#### Accuracy on go trials

The proportion between correct go trials and total go trials. Eight articles (Carriere et al., 2010; Cassarino et al., 2019; Hsieh et al., 2016; Jackson et al., 2013; Jackson & Balota, 2012; McAvinue et al., 2012; McVay et al., 2013; Mioni et al., 2019), including a total of 13 substudies, were considered in the analysis of accuracy on go trials. Carriere et al. (2010), McAvinue et al. (2012) and Mioni et al. (2019) reported only the mean and the standard deviation of omission errors (i.e., failure to respond to go stimuli), so we calculated the mean proportion of errors by dividing the mean number of omissions by the total number of go trials, separately for younger and older adults. Then, the result was subtracted from 1, since the maximum value of the accuracy index is 1 and the accuracy is complementary to error. The standard deviation of accuracy was computed by dividing the standard deviation of omission errors by the total number of go trials. Hsieh et al. (2016) reported the mean and the standard deviation of omission errors in percentages. We obtained the complementary go accuracy percentage by subtracting the mean percentage of errors from 100, and subsequently the means and the standard deviations were obtained by dividing by 100. Then, Cassarino et al. (2019) reported only the median and interquartile range (IQR) of omission errors. Hence, the authors were contacted for these data and they provided us with the means and standard deviations of this variable. Then, the values of the variable were transformed into accuracy, as in previous studies.

#### Accuracy on no-go trials

Proportion between correct no-go trials and total no-go trials. Twelve articles (Brache et al., 2010; Carriere et al., 2010; Cassarino et al., 2019; Hsieh et al., 2016; Jackson et al., 2013; Jackson & Balota, 2012; Kousaie et al., 2014; McAvinue et al., 2012; McVay et al., 2013; Mioni et al., 2019; Staub et al., 2014c; Staub et al., 2015), which included 19 substudies altogether, were considered in the analysis of accuracy on no-go trials. The study by Brache et al. (2010) did not report the standard deviation of accuracy on no-go trials, and therefore the *F*-value was considered. Carriere et al. (2010), Kousaie et al. (2014), McAvinue et al. (2012), and Mioni et al. (2019) reported only the means and the standard deviations of commission errors (false alarms to no-go stimulus). Hence, the mean proportion of errors was calculated by dividing the mean number of commissions by the total number of no-go trials and the result was subtracted from 1, since the accuracy is complementary to error and its maximum value is 1. Then, the standard deviation of accuracy was calculated by dividing the standard deviation of commission errors by the total number of no-go trials. The studies by Staub et al. (2014c) and Staub et al. (2015) reported means and standard deviations of commission errors in percentages, and we obtained these data from the graphs shown in their articles (their Fig. 1, for both) with the WebPlotDigitizer program. Again, since these studies reported the values separately for the three periods of the task, we first averaged them. Then, the complementary value of the mean commission error percentage was calculated to obtain the mean no-go accuracy in percentage, and we finally divided it and the standard deviation by 100 to have the accuracy in proportion. Staub et al. (2014c) reported the confidence intervals (95%) instead of standard deviations in the graphs, so the latter were obtained from confidence intervals through the formula $SD=\frac{ME}{t_{.025,n-1}}\times \sqrt{n}$. Also, Hsieh et al. (2016) reported means and standard deviations of no-go errors in percentage, so once again we calculated no-go accuracy as described above. Finally, Cassarino et al. (2019) reported only the median and IQR of commission errors, so the authors were contacted. They provided us with the means and standard deviations of this variable. Then, the accuracy was calculated as for previous studies.

Our study also aimed to investigate how performance changes over time in younger and older adults. However, a meta-analysis on this variable was not possible, since the minimum number of five studies was not reached. So, we will only descriptively review the results of the studies that reported block-wise performance for their experimental task.

### Risk of bias in individual studies

Only studies with healthy participants—without any psychiatric or neurological disorders—were selected. In order to assess the quality of the included studies we used the Newcastle–Ottawa Scale (NOS), a tool developed to evaluate nonrandomized studies for systematic reviews (Wells et al., 2011), and more specifically we chose a version adapted for cross-sectional studies (Patra et al., 2015). Similar to the other steps, the scoring of the NOS was performed by two authors independently, and any mismatch was solved with the intervention of a third author to reach a consensus. Details on this scale can be found in Table 3.

### Risk of bias across studies

The risk of publication bias across studies was assessed through funnel plots, provided by Meta-Essentials (Suurmond et al., 2017). In the absence of publication bias, the funnel should be symmetrical, so the studies should be equally distributed around the mean effect. With high risk of publication bias, some data are expected to be missing in the plot, leading to an asymmetrical funnel. However, this approach is limited by several factors: First of all, it is a largely subjective procedure, and in second instance there might be other causes of the funnel plot asymmetry besides publication bias (e.g., high heterogeneity among studies; Sterne et al., 2008). To partially circumvent this issue, Meta-Essentials includes a tool more specifically intended for publication bias, that is the “trim and fill” algorithm (Duval & Tweedie, 2000); this procedure imputes the potentially missing studies and calculates an unbiased estimate for the combined effect size.

### Summary measures

The difference in the mean RTs on go correct trials, accuracy on go and no-go trials between younger and older adults and interaction effects of PES were used as the summary measures.

### Synthesis of results

Four meta-analyses were performed on the SART in older and younger adults, by reporting subgroup values for each variable (RTs, PES, accuracy on go trials, accuracy on no-go trials). The two healthy subgroups were already combined in the original studies, in terms of means and standard deviations or *F* or *t* values. For each meta-analysis, the effect sizes of the individual studies and the combined effect size were estimated, reported in a forest plot, along with measures of heterogeneity (e.g., *T*), confidence and prediction intervals. Like the other “difference family” effect sizes (e.g., Cohen’s *d*, odds ratio), Hedges’ *g* is used to define the magnitude of a difference between or within groups (Van Rhee et al., 2015); this index, that applies for continuous data, is a standardized mean difference based upon a pooled and weighted standard deviation (Borenstein et al., 2009). *Heterogeneity* can be defined as the variation in the true effect sizes under a random-effects model, where it is assumed that each observed effect size estimates a different true effect (Borenstein et al., 2009). *I*^2^ and *T* are the most indicative measures of heterogeneity, the former indicating the percentage of total variation across studies due to heterogeneity versus chance and the latter representing the estimated standard deviation of true effects, so the absolute value of heterogeneity. *I*^2^ is typically interpreted as follows: 25% = low, 50% = moderate, and 75% = high (Higgins et al., 2003). The *T* value can instead be put in relation to the length of the prediction interval, which depends on it (see below for the definition of prediction interval; Borenstein et al., 2009). The confidence interval is a numerical range, centered on the point estimate of the parameter, that is likely to include the population parameter (e.g., the difference of the population means). The calculation of confidence intervals begins by setting the probability that the interval estimation does not include the parameter. Usually, 5% is accepted as the level of risk, so the confidence interval is 95% (Vaske, 2002). It is interpreted as the range that, if the parameter estimate was calculated repeatedly with different samples from the same population, it would contain the true population parameter in approximately 95% of the cases (Hoekstra et al., 2014). If the confidence interval for a difference between groups includes the zero, the result is not significant since it means that the true difference in the population might be null (Van Rhee et al., 2015). The prediction interval is based on the same (frequentist) logic, but it gives the range in which a future sampled data point might fall. Meta-Essentials calculates the prediction interval around the combined effect size, an estimate of how the true effects are distributed around the summary effect (under a random effects model; Van Rhee et al., 2015). Choosing a confidence level of 95%, the prediction interval gives the range in which the 95% of future effect sizes will fall, assuming that true effect sizes are normally distributed (Hak et al., 2016).

## Results

### Study selection

The search of PubMed, Scopus, PsycINFO and other sources (articles relevant to the topic that were cited by other articles) provided a total of 157 articles (PubMed: 61; Scopus: 45; PsycINFO: 22; other sources: 29), as shown in the PRISMA flow diagram (see Fig. 1). After discarding duplicates, 108 records remained. Titles and abstracts of the recovered articles were screened to evaluate whether they were suitable, according to the established criteria. After screening titles and/or abstracts, 61 articles were excluded. The full texts of the remaining 47 articles were examined in more detail. Of these studies, 12 were judged suitable.

### Characteristics of the studies

All 12 articles included in the meta-analytical review were published in English, and they reported the analysis on the SART, separately for younger and older adults. Nine of these used the SART version of Robertson et al. (1997), the other three used some variants instead (see Table 1). In particular, the study by Brache et al. (2010) employed a task in which participants viewed “good” or “bad” parts. Each part consisted of three black circles on a white background, one large central black circle next to two smaller circles. Participants were required to respond to the “good” part (i.e., when the larger central circle was equidistant from the others). Participants had to withhold the response when the “bad” part was shown (i.e., when the central circle was not equidistant from the others). McVay et al. (2013) used a different SART version (McVay & Kane, 2009, 2012), in which the participants had to respond to frequent nontarget words (i.e., animal names) by pressing the space bar and to rare target words (i.e., food names) by withholding the response.

**Table 1:** Summary of the studies included in the meta-analysis

	SART task	Number of go trials	Number of no-go trials	Total number of trials	Duration of the task (min.)	Variables considered
Brache et al., 2010	Modified version	950 (95%)	50 (5%)	1,000	50	RT, Accuracy no-go trials
Carriere et al., 2010	Robertson et al., 1997	200 (89%)	25 (11%)	225	4	RT, Accuracy go/no-go trials
Jackson & Balota, 2012 (Exp.1)	Robertson et al., 1997	192 (89%)	24 (11%)	216	≈4	RT, Accuracy go/no-go trials, Posterror slowing
Jackson & Balota, 2012 (Exp.2)	Robertson et al., 1997	244 (89%)	31 (11%)	275	≈5	RT, Accuracy go/no-go trials, Posterror slowing
Jackson & Balota, 2012 (Exp.3)	Robertson et al., 1997	200 (89%)	25 (11%)	225	≈10	RT, Accuracy go/no-go trials, Posterror slowing
McAvinue et al., 2012	Robertson et al., 1997	200 (89%)	25 (11%)	225	5.4	Accuracy go/no-go trials
Jackson et al., 2013 (Exp.1 Cond. 1)	Robertson et al., 1997	299 (89%)	37 (11%)	336	≈14	RT, Accuracy go/no-go trials
Jackson et al., 2013 (Exp.1 Cond. 2)	Robertson et al., 1997	299 (89%)	37 (11%)	336	≈14	RT, Accuracy go/no-go trials
Jackson et al., 2013 (Exp.2 Cond.1)	Robertson et al., 1997	299 (89%)	37 (11%)	336	≈14	RT, Accuracy go/no-go trials
Jackson et al., 2013 (Exp.2 Cond.2)	Robertson et al., 1997	299 (89%)	37 (11%)	336	≈14	RT, Accuracy go/no-go trials
McVay et al., 2013	Modified version	800 (89%)	100 (11%)	900	≈20	RT, Accuracy no-go trials, Posterror slowing
Kousaie et al., 2014 (Anglophone)	Robertson et al., 1997	200 (89%)	25 (11%)	225	NA	RT, Accuracy no-go trials
Kousaie et al., 2014 (Francophone)	Robertson et al., 1997	200 (89%)	25 (11%)	225	NA	RT, Accuracy no-go trials
Kousaie et al., 2014 (Bilinguals)	Robertson et al., 1997	200 (89%)	25 (11%)	225	NA	RT, Accuracy no-go trials
Staub et al., 2014c	Robertson et al., 1997	720 (89%)	90 (11%)	810	30	RT, Accuracy no-go trials
Staub et al., 2015	Robertson et al., 1997	720 (89%)	90 (11%)	810	30	RT, Accuracy no-go trials
Hsieh et al., 2016	Modified version	140 (70%)	60 (30%)	200	23	RT, Accuracy go/no-go trials
Cassarino et al., 2019	Robertson et al., 1997	152 (89%)	19 (11%)	171	6.48	RT, Accuracy go/no-go trials
Mioni et al., 2019	Robertson et al., 1997	200 (89%)	25 (11%)	225	4.31	RT, Accuracy go/no-go trials, Posterror slowing

*Note*. This Table displays 19 rows, although the included articles were only 12, because the study by Jackson and Balota (2012) is divided into three independent substudies, the study by Jackson et al. (2013) into four, and that by Kousaie et al. (2014) into three.

The study by Hsieh et al. (2016) employed a SART version described by Hung et al. (2013). The task was formed by a yellow, square-shaped symbol followed by a second symbol which had the same size but different color and shape. On go trials, participants had to respond to a green circular symbol by pressing a button; on no-go trials, they had to refrain this response to a red, pentagon-shaped symbol.

Although these tasks were different from the Robertson et al.' (1997) one, these studies were included because the main characteristics were comparable: the no-go condition was present, the presentation of stimuli was random or quasirandom and the typical proportions between go trials and no-go trials were respected (5% of no-go trials in Brache et al., 2010; 11% in McVay et al., 2013). Hsieh et al. (2016) presented a higher percentage of no-go trials (30%) than the other studies, but the number of no-go trials was still considerably lower than the number of go trials.

Regarding the duration of the task, some of the included studies required participants to report mind-wandering while performing the SART, so it was not possible to calculate the exact length of the task but only an approximation (as shown in Table 1).

The selected articles for the SART involved 1,522 healthy individuals, of which 832 were younger adults and 690 were older adults. The first sample included participants with a mean age of 23 years (19 and 28.25 years as the lowest and the highest mean age, respectively), the second sample a mean age of 67.98 years (mean age range: 56.2 and 77.3 years; see Table 2). The commonly used exclusion criteria included a history of neurological and psychiatric diseases, an uncorrected visual impairment, and the presence of cognitive impairment. In particular, some studies (Hsieh et al., 2016; Mioni et al., 2019) used the Mini-Mental State Examination (MMSE; Folstein et al., 1975) to investigate the presence of cognitive impairment (no dementia, MMSE > 26).

**Table 2:** Summary of demographic characteristics of the included samples

	*N* Younger	Women/ Men Younger	Age Younger (*y* ± *SD*) (Range)	Education Younger (*y* ± *SD*)	*N* Older	Women/Men Older	Age Older (*y* ± *SD*) (Range)	Education Older (*y* ± *SD*)
Brache et al., 2010	18	14/4	21 ± 1.41 (18–33)	15 **±** 1.03	17	13/4	64.29 **±** 3.08 (55–70)	13.68 **±** 2.08
Carriere et al., 2010	199	NA	24.43 ± 2.29 (20–29)	NA	43	NA	64.91 ± 4.53 (60–77)	NA
Jackson & Balota, 2012 (Exp.1)	54	29/25	19 ± .9 NA	13 ± .9	62	40/22	77.3 ± 6.9 NA	15 ± 2.5 (O. > Y., *p* < .001)
Jackson & Balota, 2012 (Exp. 2)	29	18/11	19.4 ± .8 NA	13.4 ± 1.1	38	31/7	75.8 ± 6.5 NA	14.7 ± 2.8 (O. > Y., *p* < .001)
Jackson & Balota, 2012 (Exp. 3)	31	16/15	20.9 ± 1.4 NA	14.9 ± 1.5	49	29/20	76.3 ± 6.4 NA	15.8 ± 2.6
McAvinue et al., 2012	28	18/10	28.25 ± 2.85 (20–37)	17.52 ± 1.09	27	16/11	67.78 ± 2.37 (60–75)	15.2 ± .60
Jackson et al., 2013 (Exp. 1 Cond. 1)	44	NA	25.1 ± 3.8 (18–30)	NA	27	NA	57.5 ± 5.3 (50–70)	NA
Jackson et al., 2013 (Exp. 1 Cond. 2)	45	NA	24.1 ± 3.1 (18–30)	NA	30	NA	57 ± 6.4 (50–70)	NA
Jackson et al., 2013 (Exp. 2 Cond. 1)	42	19/23	25.3 ± 3.1 (18–30)	15.1 ± 1.9	44	27/17	56.8 ± 5.6 (50–73)	15.8 ± 2.9
Jackson et al., 2013 (Exp. 2 Cond. 2)	40	22/18	25 ± 3.2 (18–30)	15.7 ± 1.9	30	21/9	56.2 ± 4.7 (50–73)	14.9 ± 2.4
McVay et al., 2013	55	NA	19.04 ± 1.79 (18–28)	12.85 ±1.32	49	NA	66.76 ± 4.35 (60–75)	15.22 ± 2.76
Kousaie et al., 2014 (Angl.)	40	25/15	21.48 ± 1.5 NA	15.55 ± 1.13	31	15/16	72.26 ± 6.43 NA	15.26 ± 2.87
Kousaie et al., 2014 (Franc.)	30	20/10	21.8 ± 2.47 NA	15.13 ± 1.38	30	23/7	72.6 ± 6.59 NA	16.2 ± 2.57
Kousaie et al., 2014 (Bil.)	51	33/18	21.49 ± 2.26 NA	15.49 ± 1.47	36	17/19	70.69 ± 5.86 NA	16.14 ± 2.85
Staub et al., 2014c	30	21/9	24.8 ± NA (18–32)	15.2 ± 2.38	30	16/14	65.2 ± NA (60–74)	14.3 ± 2.44
Staub et al., 2015	27	18/9	24.4 ± NA (18–29)	15.4 ± 2.4	25	14/11	65.5 ± NA (62–71)	14.5 ± 2.3
Hsieh et al., 2016	18	0/18	23.9 ± 2.3 (21–30)	16.3 ± 1.7	17	0/17	66.4 ± 1.2 (65–69)	16.2 ± 1.5
Cassarino et al., 2019	21	12/9	21.48 ± 7.09 NA	NA	75	42/33	68.6 ± 8.65 (60–95)	NA
Mioni et al., 2019	30	23/7	22.6 ± 4.23 (18–39)	14.17 ± 1.74	30	26/4	74.33 ± 5.54 (63–85)	14.37 ± 3.35

### Risk of bias in individual studies

The adapted Newcastle–Ottawa Scale version for cross-sectional studies scores (McPheeters et al., 2012; Table 3) showed that the included articles have a medium-low risk of bias (see Table 4).

**Table 3:** Newcastle–Ottawa Scale (adapted for cross-sectional studies)

	Q1	Q2	Q3
Selection (maximum 3 points)	Representativeness of the sample: a) Truly representative of the average in the target population (all subjects or random sampling) (1 point) b) Somewhat representative of the average in the target population (nonrandom sampling) (1 point) c) Selected group of users d) No description of the sampling strategy	Nonrespondents: a) Comparability between respondents and nonrespondents characteristics is established, and the response rate is satisfactory (1 point) b) The response rate is unsatisfactory, or the comparability between respondents and nonrespondents is unsatisfactory c) No description of the response rate or the characteristics of the responders and the nonresponders	Ascertainment of the exposure (risk factor): a) Validated measurement tool (1 point) b) Nonvalidated measurement tool, but the tool is available or described c) No description of the measurement tool
Comparability (maximum 2 points)	The subjects in different outcome groups are comparable, based on the study design or analysis. Confounding factors are controlled: a) The study controls for the most important factor (select one) (1 point) b) The study control for any additional factor (1 point)		
Outcome (maximum 2 points)	Assessment of the outcome: a) Independent blind assessment (1 point) b) Record linkage (1 point) c) Self report d) No description	Statistical test: a) The statistical test used to analyze the data is clearly described and appropriate, and the measurement of the association is presented, including confidence intervals and the probability level (*p* value) (1 point) b) The statistical test is not appropriate, not described or incomplete

**Table 4:** Quality assessment using the Newcastle–Ottawa Scale (adapted for cross-sectional studies)

				Selection		Comparability			Outcome	Total
	Q1	Q2	Q3	Quality rating	Q1	Quality rating	Q1	Q2	Quality rating	
Brache et al., 2010	b	c	a	Fair (=2)	ab	Good (=2)	a	a	Good (=2)	6
Carriere et al., 2010	b	a	a	Good (=3)	a	Fair (=1)	a	a	Good (=2)	6
Jackson et al., 2012*	b	a	a	Good (=3)	a	Fair (=1)	a	a	Good (=2)	6
McAvinue et al., 2012	b	c	a	Fair (=2)	ab	Good (=2)	a	a	Good (=2)	6
Jackson et al., 2013*	b	a	a	Good (=3)	ab	Good (=2)	a	a	Good (=2)	7
McVay et al., 2013	b	c	a	Fair (=2)	ab	Good (=2)	a	a	Good (=2)	6
Kousaie et al., 2014*	b	c	a	Fair (=2)	ab	Good (=2)	a	a	Good (=2)	6
Staub et al., 2014c	d	c	a	Poor (=1)	ab	Good (=2)	a	a	Good (=2)	5
Staub et al., 2015	d	c	a	Poor (=1)	ab	Good (=2)	a	a	Good (=2)	5
Hsieh et al., 2016	b	c	a	Fair (=2)	ab	Good (=2)	a	a	Good (=2)	6
Cassarino et al., 2019	b	a	a	Good (=3)	ab	Good (=2)	a	a	Good (=2)	7
Mioni et al., 2019	b	c	a	Fair (=2)	ab	Good (=2)	a	a	Good (=2)	6

*The substudies composing these articles were considered together, as they obtained the same NOS score.

### Synthesis of results

#### Reaction time (ms)

In the RT analysis (see Fig. 2), older adults were slower than younger adults on go trials, as indicated by a significant combined effect size (Hedges’ *g* = 1, *SE* = .13, 95% CI [.72, 1.27], 95% prediction interval [.03, 1.96], *Z* = 7.58, two-tailed *p* < .0001). There was evidence of high heterogeneity, both in terms of proportion across the observed variance (= 75.97%) and in terms of absolute value (*T* = .44), but the overall result can be considered anyway robust. Indeed, assuming that the true effects are normally distributed, we can predict that 95% of future studies will fall in the positive range between .03 and 1.96 (lower and upper limit of the prediction interval).

#### PES (ms)

**Fig. 3:**
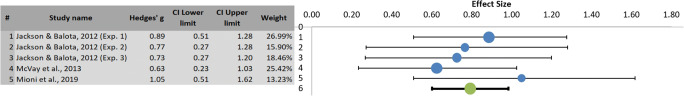
Left: Summary meta-analytical results regarding PES differences between younger and older adults, including Hedges’ *g*, confidence interval (CI), and relative weight of each study. Weight computation is explained in Fig. 2. Right: Forest plot showing the effect size (in blue) of each study with its confidence interval (in black) and the combined effect size (in green) with its confidence interval (in black) and its prediction interval (in green). The larger the blue dot, the higher the study weight. The positive effect size shows longer RTs after a commission error for older adults than for younger adults. (Color figure online)

In the PES analysis (see Fig. 3), older adults were significantly slower than younger adults after an error on no-go trials (Hedges’ *g* = .79, *SE* = .07, 95% CI [.60, .99], 95% prediction interval [.60, .99], *Z* = 11.48, two-tailed *p* < .0001). The heterogeneity proportion was null (= .00%), like the estimated standard deviations of true effects around the mean effect (*T* = .00). Thus, these results indicate no observed heterogeneity, with the important caveat of the low number of included studies.

#### Accuracy on go trials

In the accuracy on go trial analysis, older adults were numerically less accurate on go trials than younger adults, but this difference did not reach statistical significance (Hedges’ *g* = −.18, *SE* = .17, 95% CI [−.56, .19], 95% prediction interval [−1.36, 1], *Z* = −1.06, two-tailed *p* = .287), probably because of a ceiling effect in most studies. In addition, there was evidence of high heterogeneity (= 83.30%, *T* = .51).

#### Accuracy on no-go trials

**Fig. 4:**
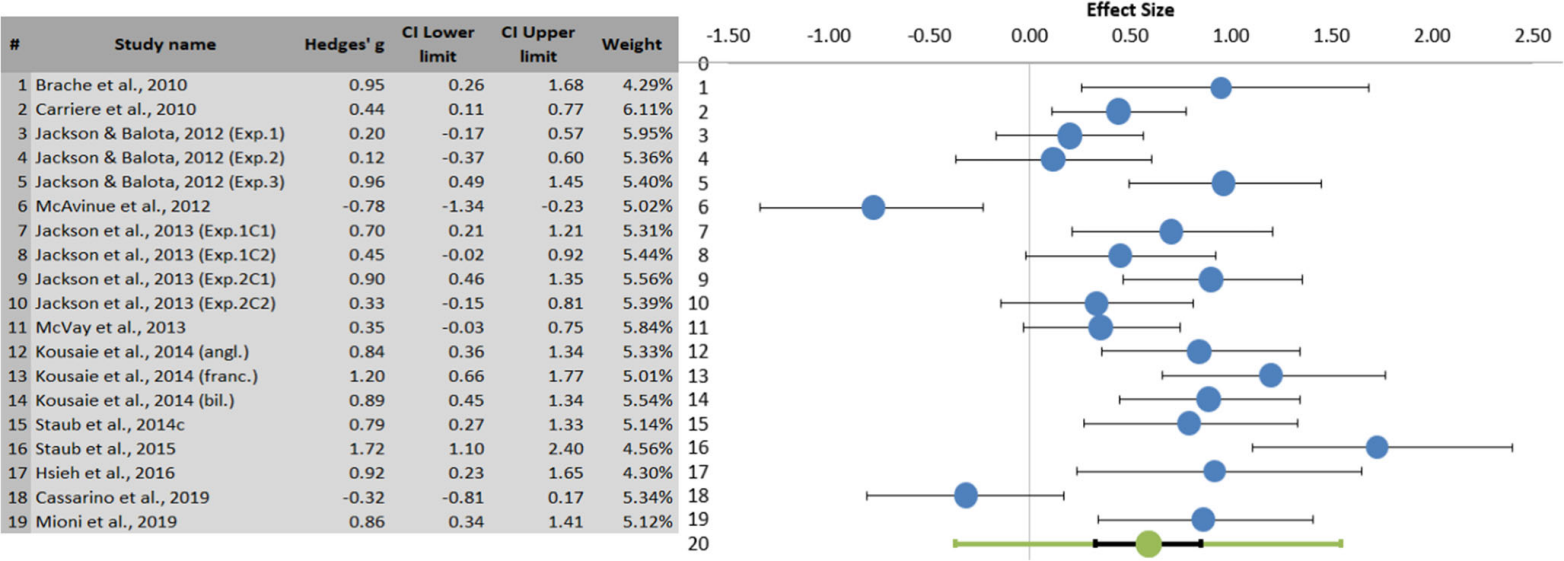
Left: Summary results of meta-analysis regarding accuracy on no-go trial differences between younger and older adults, including Hedges’ *g,* confidence interval (CI), and relative weight of each study. Weight computation as in Fig. 2. Right: Forest plot showing the effect size (in blue) of each study with its confidence interval (in black) and the combined effect size (in green) with its confidence interval (in black) and its prediction interval (in green). The larger the blue dot, the higher the study weight. The positive effect size shows higher performance in older adults than in younger adults. (Color figure online)

In the accuracy on no-go trial analysis (see Fig. 4), older adults showed significantly higher accuracy on no-go trials than younger adults (Hedges’ *g* = .59, *SE* = .13, 95% CI [.32, .85], 95% prediction interval [−.37, 1.55], *Z* = 4.69, two-tailed *p* < .0001). The heterogeneity proportion was high (= 76.77%) and the estimated standard deviation of true effect sizes was also considerable (*T* = .44). Given these high values of heterogeneity, more caution is needed when interpreting the results since, if we assume that the true effects are normally distributed, 95% of future studies will reasonably also include negative values, falling precisely between -.37 and 1.55, as indicated by the prediction interval.

#### Performance over time

Regarding the second aim of the meta-analysis (i.e., change in performance over time), as already mentioned, the cutoff established a priori (at least five studies) was not reached. Indeed, only Brache et al. (2010), Staub et al. (2014c), and Staub et al. (2015) investigated how performance on the SART varies over time. For this purpose, they divided their tasks into blocks: Brache et al. (2010) into five blocks and Staub et al. (2014c) and Staub et al. (2015) into three. As far as RTs are concerned, Staub et al. (2014c) and Staub et al. (2015) found that RTs increased in older adults between Block 1 and Block 2 (*p* < .006 and *p* < .001, in the first and second studies, respectively) and between Block 1 and Block 3 (*p* < .002 and *p* < .001, in the first and second studies, respectively), while they remained stable in younger adults.

These studies also report consistent results in terms of accuracy on no-go trials. Specifically, the commission errors increased in younger adults over time (Brache et al., 2010; differences between Block 1 and Block 2 *p* < .004 and *p* < .007, and between Block 1 and Block 3 *p* < .003 and *p* < .009 in Staub et al., 2014c and Staub et al., 2015, respectively). On the contrary, they decreased (differences between Block 1 and Block 2 *p* < .001 in Staub et al., 2014c; between Block 1 and Block 2 and between Block 1 and Block 3 *p* < .001 in Staub et al., 2015) or remained stable (Brache et al., 2010) in older adults.

### Risk of bias across studies

**Fig. 5:**
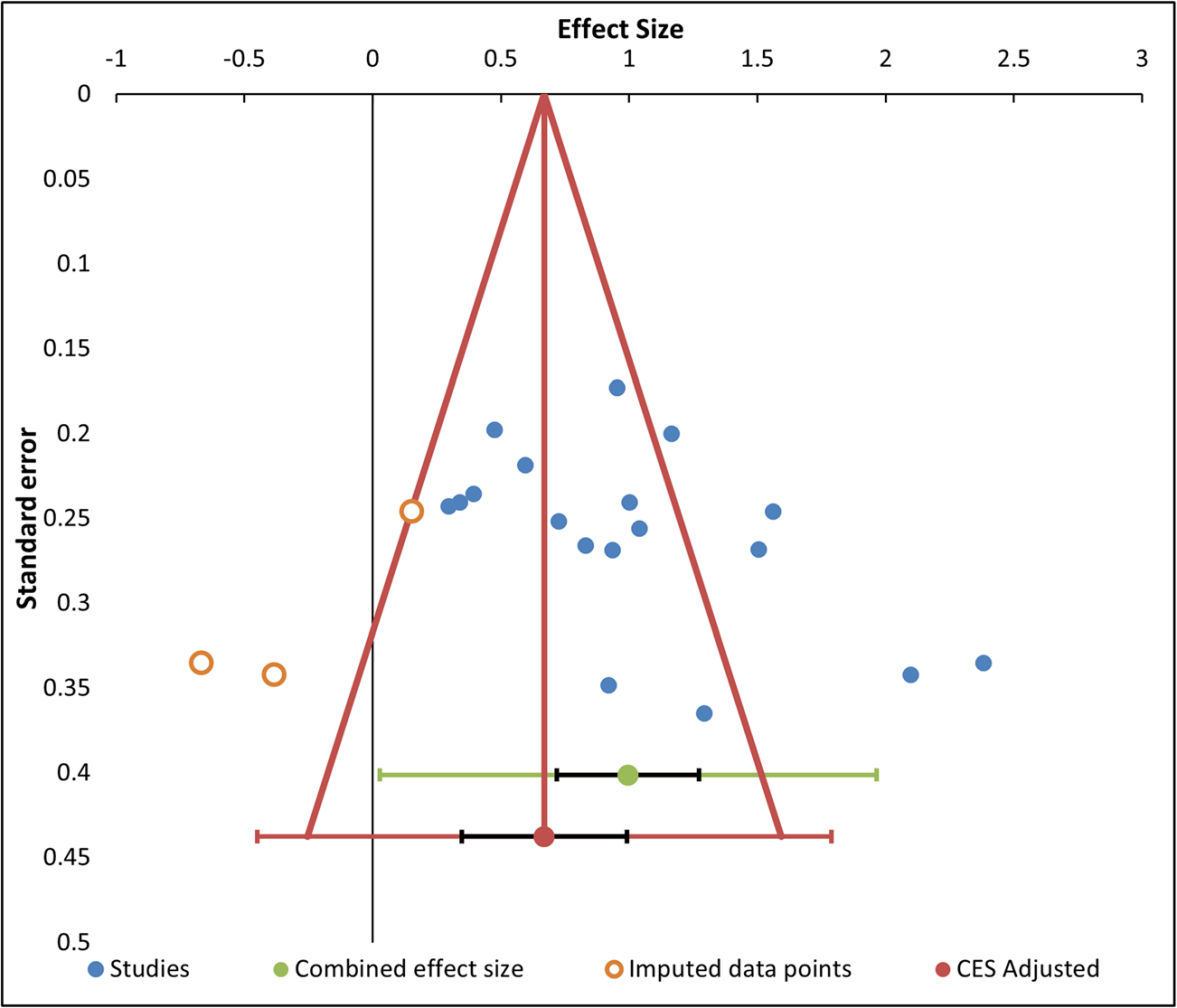
Funnel plot of the studies in the RTs analysis, represented by blue dots, with effect size (*x*-axis) and standard error (*y*-axis). There is also the combined effect size (green dot) with its confidence interval (black) and prediction interval (green), and the adjusted effect size (red dot) for imputed data points with the corresponding intervals (black and red, respectively). The adjusted effect size is lower than the original one because it takes into account three missing studies located on the left of the mean effect. (Color figure online)

Regarding the risk of bias across studies for the RTs analysis, the funnel plot (see Fig. 5) shows some asymmetry among the studies with higher standard errors (at the bottom of the graph), which are all distributed on more positive values than the mean effect. This subjective statement is partially confirmed by the results of the tests for funnel plot’s asymmetry (Egger’s test and Begg and Mazumdar test), with only the first being significant (*t* = 2.68, *p* = .016, and *z* = 1.86, *p* = .063, respectively). This asymmetry could be due to publication bias, as the “trim and fill” method found three missing studies on the left side of the mean effect. Therefore, the adjusted combined effect size when considering the imputed data points is lower (Hedges’ *g* = .67) than the original one (Hedges’ *g* = 1.00), but still significant (95% CI [.35, .99]).

**Fig. 6:**
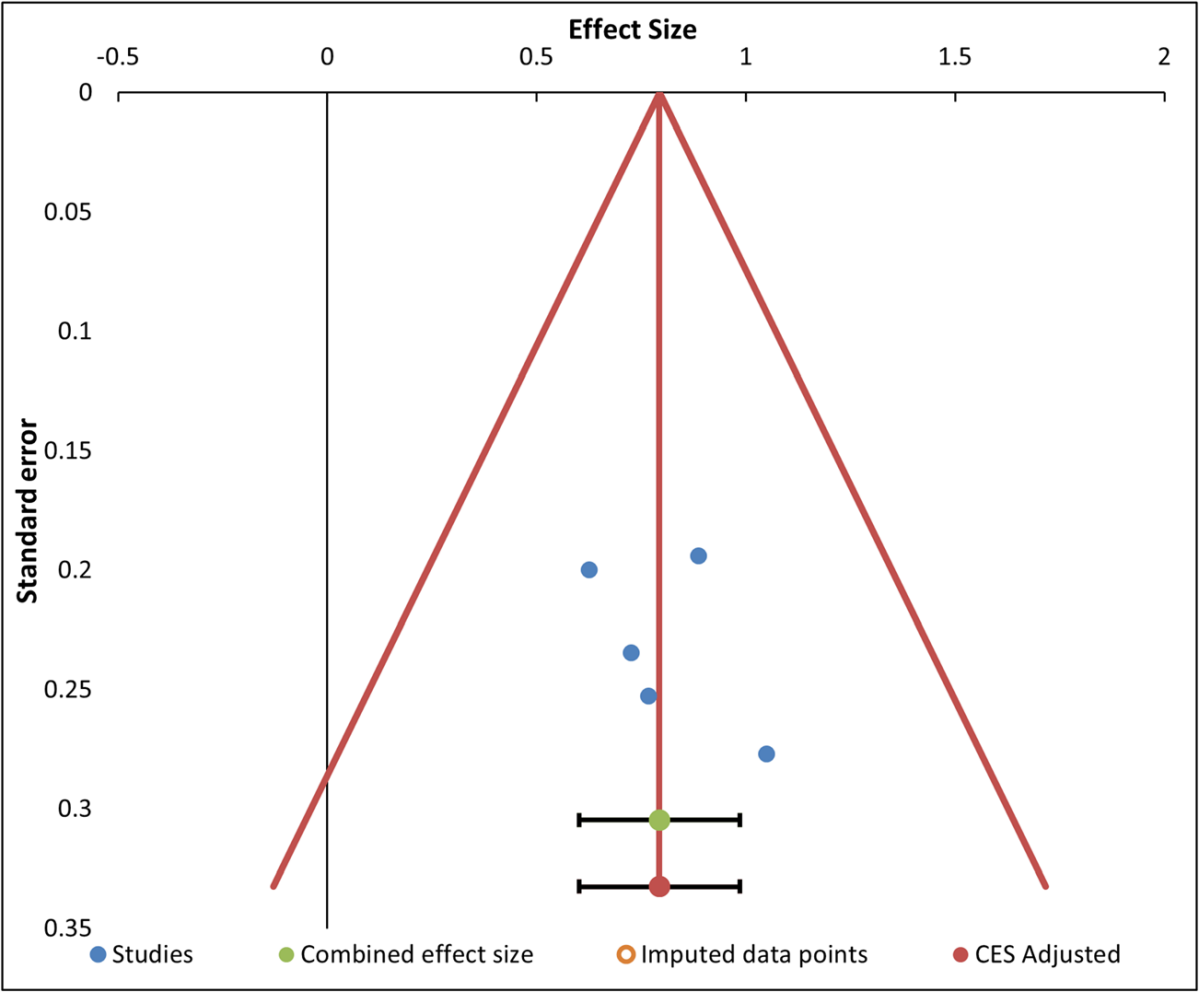
Funnel plot of the studies in the PES analysis, represented by blue dots, with effect size (*x*-axis) and standard error (*y*-axis). The plot also reports the combined effect size (green dot) and the adjusted effect size (red dot) with their confidence intervals (black) and prediction intervals (green and red, respectively). The original combined effect size is equal to the adjusted one since the “trim and fill” method found no missing studies. (Color figure online)

The approaches for the evaluation of publication bias should however be used only when there is a reasonable number of studies (at least 10; Borenstein et al., 2009; Sterne et al., 2008). Therefore, the funnel plot for PES analysis (see Fig. 6) is difficult to interpret due to the paucity of studies. Considering this caveat, no evidence of asymmetry arises from the Egger’s test (*t* = .83, *p* = .47) and the Begg and Mazumdar test (*z* = .98, *p* = .33). Moreover, the “trim and fill” method found no missing studies, suggesting no evidence of publication bias.

**Fig. 7:**
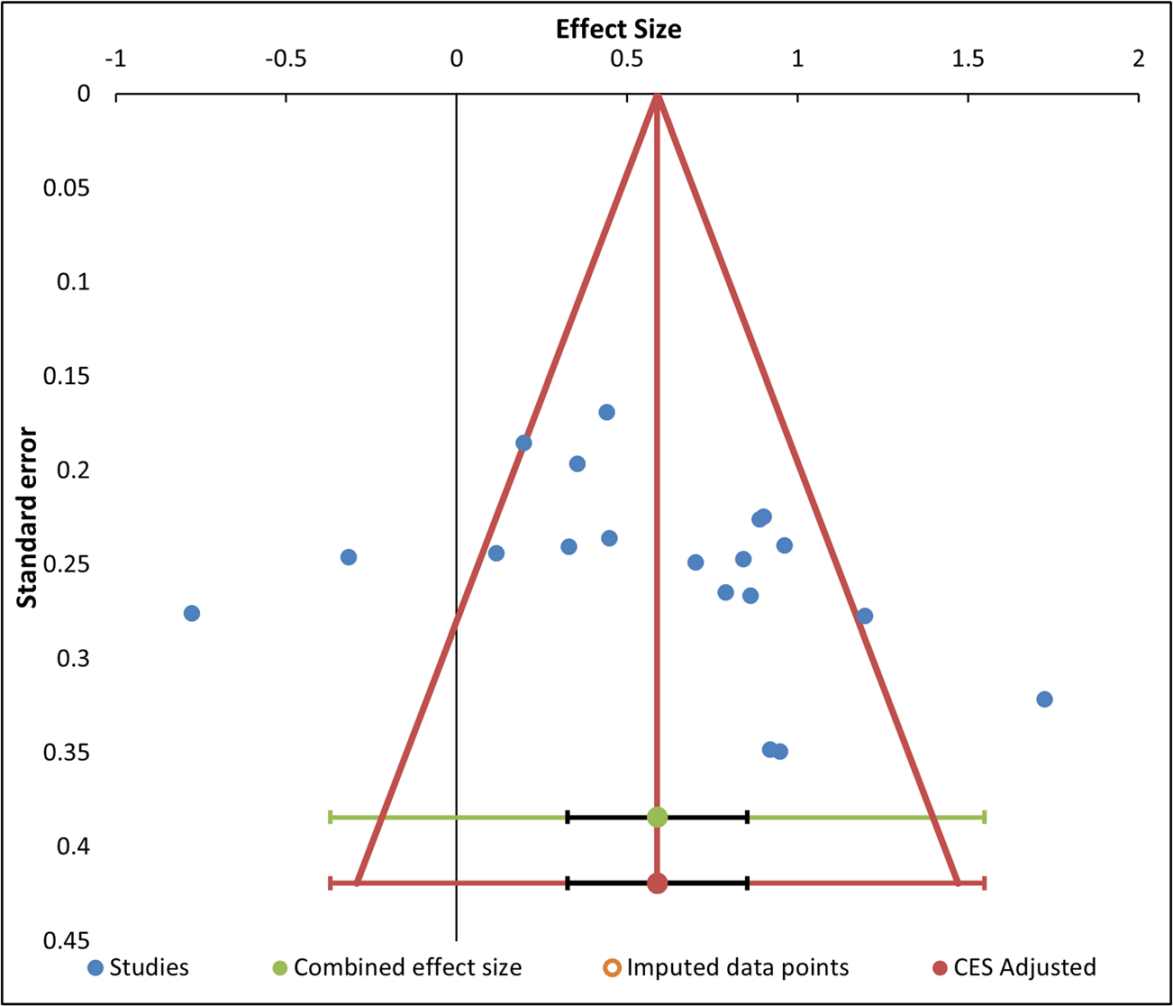
Funnel plot of the studies in the no-go accuracy analysis, represented by blue dots, with effect size (*x*-axis) and standard error (*y*-axis). The combined effect size (green dot) and its adjusted estimate (red dot) are also depicted, with their confidence intervals (black) and prediction intervals (green and red, respectively). The two combined effects are equal since the “trim and fill” algorithm found no evidence of publication bias. (Color figure online)

The funnel plot for no-go accuracy (Fig. 7) does not show relevant asymmetry, as the studies are more or less equally distributed around the mean effect. Indeed, the Egger’s test and the Begg and Mazumdar test were both not significant (*t* = 1.60, *p* = .189 and *z* = 1.15, *p* = .436, respectively). In addition, the “Trim and Fill” algorithm found no missing studies, suggesting no asymmetry due to publication bias.

## Discussion

The aim of the present meta-analytical study was to evaluate age-related differences in sustained attention, using the SART as the most representative task to measure this construct. Overall, meta-analytical evidence showed that older adults were slower than younger adults in responding to go stimuli and after an error on no-go trials; nevertheless, older adults outperformed younger adults in terms of accuracy on no-go trials, while the two age groups did not differ in terms of accuracy on go trials.

### Age-related slowing and increased accuracy rate

The age-related slowing found in our meta-analysis confirms a robust trend present in literature: an increase in RTs and/or RT variability with age in different cognitive tasks (e.g., Der & Deary, 2006; Dykiert et al., 2012; Salthouse, 1996), including attentional tasks (e.g., Fortenbaugh et al., 2015; Lufi & Haimov, 2019). Several alternative explanations have been proposed to describe this effect.

From an anatomical perspective, this decline in speed has been mainly attributed to the age-related deterioration of the white matter, that leads to a reduction in the efficiency of communication between brain regions (disconnection hypothesis; O’Sullivan et al., 2001); also, neural measures not obviously linked to connection efficiency, like the brain volume, have been found to be related to measures of speed (see Salthouse, 2017, for a review). In relation to that, the speed deficit theory asserts that the cognitive problems faced by older adults are rooted in a slowing down of the brain’s processing systems (Salthouse, 1996).

Another explanation, that is not mutually exclusive, for the RTs increase observed in older adults concerns an age-related difference in speed–accuracy trade-off (Hertzog et al., 1993; Smith & Brewer, 1995), which may also account for the higher accuracy on no-go trials. Indeed, older adults may have adopted a more conservative and controlled response strategy while performing the task, emphasizing accuracy over speed, while younger adults may have prioritized more response speed, thereby being more error prone on no-go trials (Fortenbaugh et al., 2015; Staub et al., 2013). Similarly, also, the age-related increase in PES could be considered as a further indicator of this increased cautiousness.

According to the *diffusion model* approaches (see Ratcliff & Smith, 2004, for a review), older adults typically need to collect more evidence before selecting a response compared with their younger counterparts (Ratcliff et al., 2004; Starns & Ratcliff, 2010). Moreover, evidence exists of an age-related increase in the response criterion (Criss et al., 2014), a parameter of the signal detection theory, which represents the willingness of the subject to report a signal in ambiguous conditions; the higher the criterion, the higher the evidence the subject requires to report a signal, indicating a more conservative strategy.

This more prudent strategy could lead older adults to better inhibit responses to no-go stimuli, in line with studies demonstrating preserved inhibitory abilities in older adults during go/no-go procedures (Grandjean & Collette, 2011; Staub et al., 2014b). Indeed, the SART could be more precisely conceptualized as a compound measure of inhibitory control and sustained attention rather than a pure measure of the latter (Carter et al., 2013; Stevenson et al., 2011). Therefore, this meta-analysis challenges the notion of general inhibition deficits in older adults (Hasher & Zacks, 1988; Healey et al., 2008), in line with recent reports that found inconsistent results or no evidence of age-related deficits in inhibition capacities (Rey-Mermet & Gade, 2018).

The controlled strategy adopted by older adults could lead to a better inhibition not only of task-related contents but also internally generated irrelevant stimuli (e.g., task-unrelated thoughts; TUTs), as demonstrated by the lower amount of mind-wandering in aging during sustained attention tasks (Fountain-Zaragoza et al., 2018; Giambra, 1989; Jackson et al., 2013; Jackson & Balota, 2012; McVay et al., 2013; Staub et al., 2014b, 2014c; Staub et al., 2015). The reduced amount of mind-wandering exhibited by older adults could be explained by a higher degree of control over the task coupled with an increased task difficulty, when compared with younger adults (Smallwood, 2015). The lower tendency to mind-wander in older adults could also be due to the higher motivation and interest they typically show in cognitive tasks when volunteering in lab studies (Staub et al., 2014b, 2014c; Staub et al., 2015; Thackray & Touchstone, 1981), which would help them to endogenously maintain sustained attention over the task.

From the studies included in the systematic search, five of them analyzed the mind-wandering effects during the execution of the SART. Of those studies, three included mind-wandering probes (McVay et al., 2013; Jackson et al., 2012; Jackson et al., 2013), while in the other two studies, participants were asked to fill a questionnaire after the task (Staub et al., 2014c; Staub et al., 2015); thereby, it was not possible to perform a meta-analysis, due to the paucity of available studies with a consistent approach. Despite methodological differences in the employed tasks (i.e., with and without mind-wandering probes), in all five studies it was found that older adults tend to mind-wander less frequently than younger adults. Different data were found when a particular mind-wandering category was taken into consideration: “task-related interference” (TRI; e.g., Smallwood et al., 2004). TRI differs conceptually from “task-unrelated thoughts” (TUT; used in previous studies), because it refers to task-related thinking, but both are associated with higher go/no-go errors (McVay & Kane, 2012). McVay et al. (2013) evaluated TRI and showed that older adults experienced more TRI than younger people. However, younger adults reported a higher total mind-wandering (21% of TRI and 51% of TUT, for a total of 72%) than older adults (31% of TRI and 17% of TUT, for a total of 48%). In previous studies, the absence of TRI as a response may have inflated age differences in the rate of mind-wandering.

Moreover, McVay et al. (2013) found that when the level of mind-wandering was taken into account, age-difference between groups on the SART disappeared, indicating that older adults outperformed younger ones partially because of their reduced mind-wandering. Jackson et al. (2013) examined self-reported and probe-caught mind-wandering in two different experiments but they did not directly compare performance between tasks. However, they suggest that older adults might find the SART more difficult (in both experiments) and more interesting (in the probe-caught version), thus reducing their mind-wandering. It is important to remember that these age-related differences in mind-wandering have been shown to be partially due to age-related differences in motivation (Seli et al., 2017; Seli et al., 2020; Staub et al., 2015).

Although some of the included studies measured interest or motivation of the participants (Jackson et al., 2013; Jackson & Balota, 2012; Staub et al., 2014c; Staub et al., 2015), it was not possible to meta-analytically assess their influence on SART performance among younger and older adults, since in both cases the threshold of a minimum number of five studies was not reached. Moreover, given that these dimensions were measured in heterogeneous ways, it was not reasonable to include them in a single meta-analysis.

Nevertheless, from a descriptive perspective, older adults were generally more motivated before performing the task (Staub et al., 2014c; Staub et al., 2015) or found it more interesting (see Experiments 2 and 3 in Jackson & Balota, 2012, and Experiment 2 in Jackson et al., 2013) than younger adults. This age difference could be explained by the fact that younger adults were in most cases university students (Brache et al., 2010; Cassarino et al., 2019; Jackson & Balota, 2012; McVay et al., 2013; Mioni et al., 2019), thus highly familiar with the context and the experience of these studies in contrast with older adults, for whom the novelty effect could explain their higher degree of motivation and/or interest. Moreover, personality traits like conscientiousness, which is higher in older adults (see Experiment 1 and 2 in Jackson & Balota, 2012), could partially explain this difference, because older adults were more likely to take the task seriously.

This evidence provides also support to the *mindlessness theory of vigilance* (Manly et al., 1999; Robertson et al., 1997), according to which failures on sustained attention tasks are caused by mindlessness, a state induced by the monotony of the task in which attention is disengaged from task-related stimuli and captured by task-unrelated ones. Since older adults are more intrinsically motivated and adopt a more controlled strategy, they are less likely to experience task-unrelated thoughts (Staub et al., 2013). On the other hand, according to the *resource account* (Warm et al., 2008), vigilance performance is dependent upon variations in attentional resources; thus, if we assume that aging is associated with a resource deficit, older adults should perform worse than younger adults on sustained attention tasks (Craik & Byrd, 1982). However, besides performing better than younger adults, older adults do not differ from them in terms of workload ratings related to the task (Staub et al., 2014b, 2014c). Standard sustained attention tasks might not be demanding enough to over-tax the reduced attentional capacities of older adults (Thomson & Hasher, 2017), and this is suggested by the fact that under more demanding conditions (e.g., perceptually degraded stimuli, faster presentation of stimuli) some age differences arise (Mouloua & Parasuraman, 1995; Parasuraman et al., 1989).

In the neuroimaging literature, a more controlled response strategy has been associated with a higher activation of multiple regions, among which a key role is played by the anterior cingulate cortex (ACC) and the lateral prefrontal cortex (PFC). The activation of those regions during top-down control leads to enhanced attention on relevant task-information (Hester et al., 2004; Simoes-Franklin et al., 2010). In the aging brain, studies showed that older adults increase activity in the ACC on go/no-go tasks (Hester et al., 2004; Nielson et al., 2002) and engage the lateral PFC with time on task, indicating the involvement of higher cognitive control and improvement in performance over time (Sharp et al., 2006). Also, ERP studies on the SART (Staub et al., 2014b; Staub et al., 2015) showed that, when compared with younger adults, older adults exhibit a higher P3 amplitude to nontargets and a higher P2 amplitude over frontocentral electrodes regardless of the type of stimulus (go, no-go), indicating a higher allocation of top-down attentional resources throughout the duration of the task.

Concerning the second aim of the study—namely, assessing change in performance over time—we could not include enough studies to be able to perform a meta-analysis. However, the identified studies showed that as the task goes on, older adults show increased RTs and enhanced accuracy compared with younger adults (Brache et al., 2010; Staub et al., 2014c; Staub et al., 2015), with no effect of fatigue, when considered. Indeed, this time-on-task pattern suggests that older adults had longer RTs along the task not (only) because the task was too demanding, but to actually increase the performance level in terms of accuracy. This effect might be linked to the fact that older adults are greatly motivated to perform the task proficiently, have less intrusive thoughts, which might allow them to focus on the task, maintaining a high level of attention without habituation. Thus, as the task goes on, they might prefer to shift towards greater accuracy at the expenses of speed.

### Age-related posterror slowing increase

Our meta-analysis found an increased PES in older adults, a result reported also by other studies in the literature (Band & Kok, 2000). Different accounts, either adaptive or maladaptive, have been proposed regarding this phenomenon (see Danielmeier & Ullsperger, 2011, for a review); however, the functional role of PES is still largely debated. According to the cognitive control account, this kind of posterror adjustment would reflect the activation of the performance monitoring system, as suggested by the positive correlation between PES and the error-related activity in posterior medial frontal regions found in functional magnetic resonance imaging (fMRI) and electroencephalography (EEG) studies (Danielmeier & Ullsperger, 2011), hence indicating the implementation of cognitive control after an error. Given the correlation between PES and activity in performance monitoring structures, an increased slowing after an error could indicate a higher recruitment of cognitive control in the elderly (Staub et al., 2014c).

Other accounts propose alternative explanations for the PES, as only a few studies have shown an association between PES and increased posterror accuracy, but most of the time the two variables are not correlated. After an error, decision boundaries change (as shown by drift diffusion models; Purcell & Kiani, 2016; Ullsperger & Danielmeier, 2016) and early posterror adjustments might reflect a general orienting reflex related to the infrequency of the events, rather than increased cognitive control (Notebaert et al., 2009). Further, according to Smallwood et al. (2004), PES may reflect a type of task-related mind-wandering, also called task-related interference (TRI). When an error is detected, the participant initiates a type of reactive process that may include self-evaluation of performance. Since older adults are typically more interested and motivated when performing a task than younger adults, they may be more likely to engage in these task-related thoughts after realizing they made an error, which could explain their disproportionate posterror slowing on the SART (Jackson & Balota, 2012). This hypothesis is not necessarily in contrast with the idea of a greater engagement of cognitive control processes in older adults, since these evaluative thoughts can be seen as the expression of higher attentiveness to the task, aimed at adjusting subsequent performance (Staub et al., 2013).

Similar to the interpretations provided for the slowing in the go trials, another explanation of the increase in PES might be related to a further indicator of the enhanced cautiousness in aging (Dutilh et al., 2012; Fortenbaugh et al., 2015).

### SART characteristics and age-related changes

A previous review on aging and sustained attention (Staub et al., 2013) suggested that the inconsistency in the sustained attention literature may arise from the heterogeneity of methods applied to measure it, and the present meta-analysis provides support to this perspective.

For this reason, we have included studies with a SART-like paradigm (Robertson et al., 1997), excluding all those that used a fixed, predictable sequence and frequent no-go stimuli. In our meta-analysis, we found that, in SART and SART-like paradigms (high-frequency go trials), older adults may overcome their younger counterparts at least for accuracy to no-go stimuli, while in previous reports on traditional formatted tasks (TFTs; low-frequency go trials) an opposite pattern was found (Staub et al., 2013). According to Staub and colleagues, this is because sustained attention is the result of the interaction between top-down and bottom-up processes, which could be both differentially affected by aging and involved by the two types of tasks. The performance on SART and SART-like paradigms may depend more on self-sustained attention and top-down/controlled processing, since it requires to overcome a habitual response that has become automatic, while TFTs may rely more upon bottom-up processes. Hence, the controlled strategy exhibited by older adults, also promoted by a higher degree of interest and motivation, could explain their better performance on this type of task. On the other hand, the age-related decline in bottom-up attentional and sensory processes (Lee et al., 2018; Lindenberger & Baltes, 1994) could explain the worse performance by older adults on TFTs. An ERP study (Staub et al., 2015) demonstrated that also on a TFT, older adults tend to exert higher cognitive control than younger adults. Therefore, another hypothesis is that maintaining this strategy over the task could have opposite effects based on the task type, being too effortful and thus detrimental on TFTs and effective on SART and SART-like paradigms (Staub et al., 2015).

Importantly, it should be noted that, in order to be included in the meta-analysis, the studies had to satisfy some inclusion criteria such as using a SART paradigm with a lower percentage of no-go than go trials, being tested in healthy younger and older adults, and providing enough rigorous statistical information to be included in the meta-analysis. After the screening, 12 studies were considered suitable for the meta-analysis and, of those, 10 studies showed consistent evidence in one direction (i.e., longer RTs and increased accuracy in older adults compared with younger adults, in no-go trials). Thus, it is noteworthy that some of the studies that found opposite or mixed findings in the literature could have been left out from the meta-analysis because they did not meet the inclusion criteria.

### Future directions

The findings of the present meta-analysis suggest many developments for future aging-related research on the SART. An interesting direction would be to explicitly manipulate speed–accuracy trade-off and motivation (e.g., by providing feedback/rewards during the task or manipulated task instructions), to test the hypothesis of a crucial role of these aspects when considering age differences in performance on the SART. Future studies should also control for individual differences in speed–accuracy trade-off by computing a skill index that accounts for both accuracy and RTs (e.g., Saucedo Marquez et al., 2013; Seli, 2016), in order to obtain a purer measure of participant’s efficiency on the SART and to assess whether this composite measure actually changes with age.

Moreover, since we found an insufficient number of studies that investigated changes of sustained attention over time in aging, there was not enough evidence to perform a meta-analysis; hence, more future studies should investigate whether and how sustained attention changes over time and whether older adults show a more consistent level of performance during the task than younger adults do (e.g., by dividing the task into blocks or by single-trial analysis).

A promising future avenue could also be to investigate age-related differences in neurophysiological correlates of the SART. Previous EEG studies on younger adults found that adaptation after attention lapses (related to PES) is associated with decreased posterior alpha and increased frontal theta activity (van Driel et al., 2012). Future studies could unveil whether older adults show similar EEG patterns during PES, possibly reflecting the recruitment of additional brain networks with respect to younger adults.

Finally, research on aging and SART could be further expanded for clinical purposes. Recent trends in clinical neuropsychology showed the great potential of computerized testing to detect subtle impairments and rehabilitate neurological conditions (Bogdanova et al., 2016; Kueider et al., 2012). The SART, and its consistent age-related pattern, could be exploited to identify individuals with vigilance failures, and performance on the SART could be a potential marker of cognitive decline (Fortenbaugh et al., 2017). This could be further developed by combining behavioral performance with EEG indices (such as P3; Porcaro et al., 2019), to exploit multimodal biomarkers of cognitive decline.

### Limitations

There are some limitations to consider in this meta-analytical study. First, the relatively low number of included studies prevented us from analyzing other variables which could have given a broader view of sustained attention in aging. Indeed, due to paucity of studies, it was impossible to investigate the second question of this study: the change of attentional performance over time. This limitation also affected the PES analysis, since only five studies were considered. We also have to note that, in the PES analysis, the data used to compute the effect sizes are drawn not from a simple contrast analysis, but from an interaction effect (i.e., Age × No-Go Response Type). This less direct index requires more caution when interpreting the results related to the increased PES in older adults.

Another important limitation was the high heterogeneity of the included studies, which limits the strength of the results, particularly in the analysis of no-go accuracy. Many factors could have contributed to this heterogeneity, including the age range of the included participants that considerably differed across studies (particularly for older adults), or the characteristics of the task. Not less important to consider is the result of the quality assessment: using a modified version of NOS scale, the majority of studies was rated as of “fair quality” (i.e., with a total score of 5 or 6), and only two were ranked as “good quality” studies (i.e., with a score of 7 or more). Higher quality studies are desirable in the future.

## Conclusions

The present meta-analytical study expands the knowledge on the age-related differences in the domain of sustained attention, and supports the idea that cognitive aging is a complex, multifaceted phenomenon, not unequivocally associated with decline. Indeed, in accordance with our hypothesis, older adults show good performance on the SART, with increased accuracy on no-go trials (despite longer RTs) compared with younger adults. These results could be explained by a different use of attentional resources by older adults with respect to younger ones: on the one hand, older adults may adopt a controlled, top-down response strategy that trades speed for accuracy. Further, they might show good performance for other reasons that are not necessarily mutually exclusive (e.g., higher motivation, reduced mind-wandering, greater fear of evaluation), but that could also require greater cognitive effort. On the other hand, younger adults may rely upon a more automatic responding mode, with higher speed but also a higher likelihood of commission errors.

This meta-analysis provides a systematic and quantitative overview of sustained attention abilities in aging. Further, our work identifies the need to investigate age differences over time more in depth, also considering individual aspects (e.g., mind-wondering, motivation, fatigue) as factors which may play a key role in task performance. Given the importance of sustained attention for general cognitive functioning in life, this quantitative analysis highlights solid results as well as points that need further testing, providing a basis for future directions in aging research.
